# Ribosome-associated quality control of membrane proteins at the endoplasmic reticulum

**DOI:** 10.1242/jcs.251983

**Published:** 2020-11-27

**Authors:** Ben P. Phillips, Elizabeth A. Miller

**Affiliations:** MRC Laboratory of Molecular Biology, Cambridge CB2 0QH, UK

**Keywords:** Endoplasmic reticulum, Membrane protein, Protein folding, Ribosome, Translation

## Abstract

Protein synthesis is an energetically costly, complex and risky process. Aberrant protein biogenesis can result in cellular toxicity and disease, with membrane-embedded proteins being particularly challenging for the cell. In order to protect the cell from consequences of defects in membrane proteins, quality control systems act to maintain protein homeostasis. The majority of these pathways act post-translationally; however, recent evidence reveals that membrane proteins are also subject to co-translational quality control during their synthesis in the endoplasmic reticulum (ER). This newly identified quality control pathway employs components of the cytosolic ribosome-associated quality control (RQC) machinery but differs from canonical RQC in that it responds to biogenesis state of the substrate rather than mRNA aberrations. This ER-associated RQC (ER-RQC) is sensitive to membrane protein misfolding and malfunctions in the ER insertion machinery. In this Review, we discuss the advantages of co-translational quality control of membrane proteins, as well as potential mechanisms of substrate recognition and degradation. Finally, we discuss some outstanding questions concerning future studies of ER-RQC of membrane proteins.

## Introduction

Eukaryotic cells can expend up to 30% of their energy resources on the production of new proteins (Buttgereit and Brand, 1995). One reason that protein synthesis accounts for such a large proportion of cellular energy consumption is the low yield of functional proteins and protein complexes. It is estimated that between 10 and 30% of nascent peptides in mammalian cells are degraded during or shortly after synthesis as a result of defects in biogenesis or maturation (Schubert et al., 2000; McShane et al., 2016). The consequences of synthesis errors can be severe; aberrant proteins resulting from improper biogenesis tend to aggregate, which in turn is associated with cellular damage. This can result in diseases such as Alzheimer’s disease, Huntington’s disease, Parkinson’s disease, and some forms of type II diabetes, lung cancer and heart disease (Hartl, 2017). Substitution of even a single amino acid that influences the topology of the non-essential prion protein (PrP) can cause protein aggregation at the cellular level and neurodegeneration at the organismal level (Hegde et al., 1998).

In this Review, we will discuss how defects in biogenesis of membrane proteins (MPs) are handled early during their synthesis while they are still associated with translating ribosomes. Misfolding mutations in MPs have been associated with retinal degeneration (Cremers et al., 2020), cystic fibrosis (CF) (Kim and Skach, 2012), Charcot–Marie–Tooth disease (Bergoffen et al., 1993) and many other pathologies. Of the 1100 MPs annotated in the UniProt disease-related database, it has been hypothesised that most disease-causing mutations result in misfolding and/or aggregation (Marinko et al., 2019). In order to avoid cellular toxicity associated with aberrant or misfolded MPs, eukaryotic cells have evolved an extensive quality control network.

The central hub of MP quality control is the endoplasmic reticulum (ER). It is a major site of MP synthesis, and monitors various physical and contextual properties, including folding state (Vembar and Brodsky, 2008), glycosylation (Lamriben et al., 2016), oligomerisation (Natarajan et al., 2019) and organelle localisation (Chen et al., 2014; Okreglak and Walter, 2014; Weir et al., 2017). The cornerstones of ER quality control include the soluble chaperones of the cytosol and ER lumen that promote protein folding; the unfolded protein response (UPR) (Karagöz et al., 2019), a global cellular response to accumulation of misfolded proteins; and ER-associated degradation (ERAD), which disposes of terminally misfolded proteins (Wu and Rapoport, 2018). These and other related quality control mechanisms have been extensively reviewed recently (Juszkiewicz and Hegde, 2018; Phillips et al., 2020) and will not be covered here. Instead, we will focus on quality control that acts during translation to prevent the accumulation of aberrant MPs at the earliest opportunity. This pathway has parallels with cytoplasmic ribosome-associated quality control (RQC) and has thus been termed ER-RQC (Joazeiro, 2019). In the context of aberrant MPs, ER-RQC appears to act pre-emptively to avoid the accumulation of potentially toxic misfolding MPs (Lakshminarayan et al., 2020; Trentini et al., 2020). Notably, unlike canonical RQC, this newly discovered co-translational quality control does not appear to respond to errors in the mRNAs encoding substrates. Instead, the process responds to the biogenesis state of the substrate. In this Review, we will discuss how defects in MP biogenesis might arise and describe the basic machinery involved in RQC. We will subsequently explore the relationship between ER-RQC and aberrant MPs, and describe potential mechanisms that might trigger ER-RQC, before considering outstanding questions in the field.

## Biogenesis of MPs

The majority of MPs follow a common biogenesis pathway that has been heavily studied and is, for the most part, well understood (Rapoport et al., 2004; Osborne et al., 2005; Shao and Hegde, 2011; Park and Rapoport, 2012; Barlowe and Miller, 2013; Cymer et al., 2015b; Rapoport et al., 2017). Substrates containing either an N-terminal signal peptide (SP) or a hydrophobic transmembrane-domain (TMD) are engaged at the mouth of the ribosome exit tunnel by the signal recognition particle (SRP). The SRP-bound ribosome is recruited to the ER membrane via interactions between SRP and its cognate receptor (SRP receptor, or SR). The nascent chain is subsequently transferred to the trimeric Sec61 translocon complex in an incompletely understood molecular hand-off reaction (Jomaa et al., 2017; Kobayashi et al., 2018).

In the case of SP-containing proteins, the SP intercalates at the Sec61 lateral gate, propping open the channel and allowing passage of the nascent chain into the lumen (Voorhees and Hegde, 2015). The SP is subsequently proteolytically cleaved and removed. If the first hydrophobic element is a TMD, this sequence intercalates at the lateral gate of the translocon, before partitioning into the lipid bilayer. Following insertion of the first TMD, subsequent TMDs are sequentially inserted by the Sec61 translocon with soluble regions alternating between the cytosol and the lumen (Rapoport et al., 2004).

**Fig. 1:**
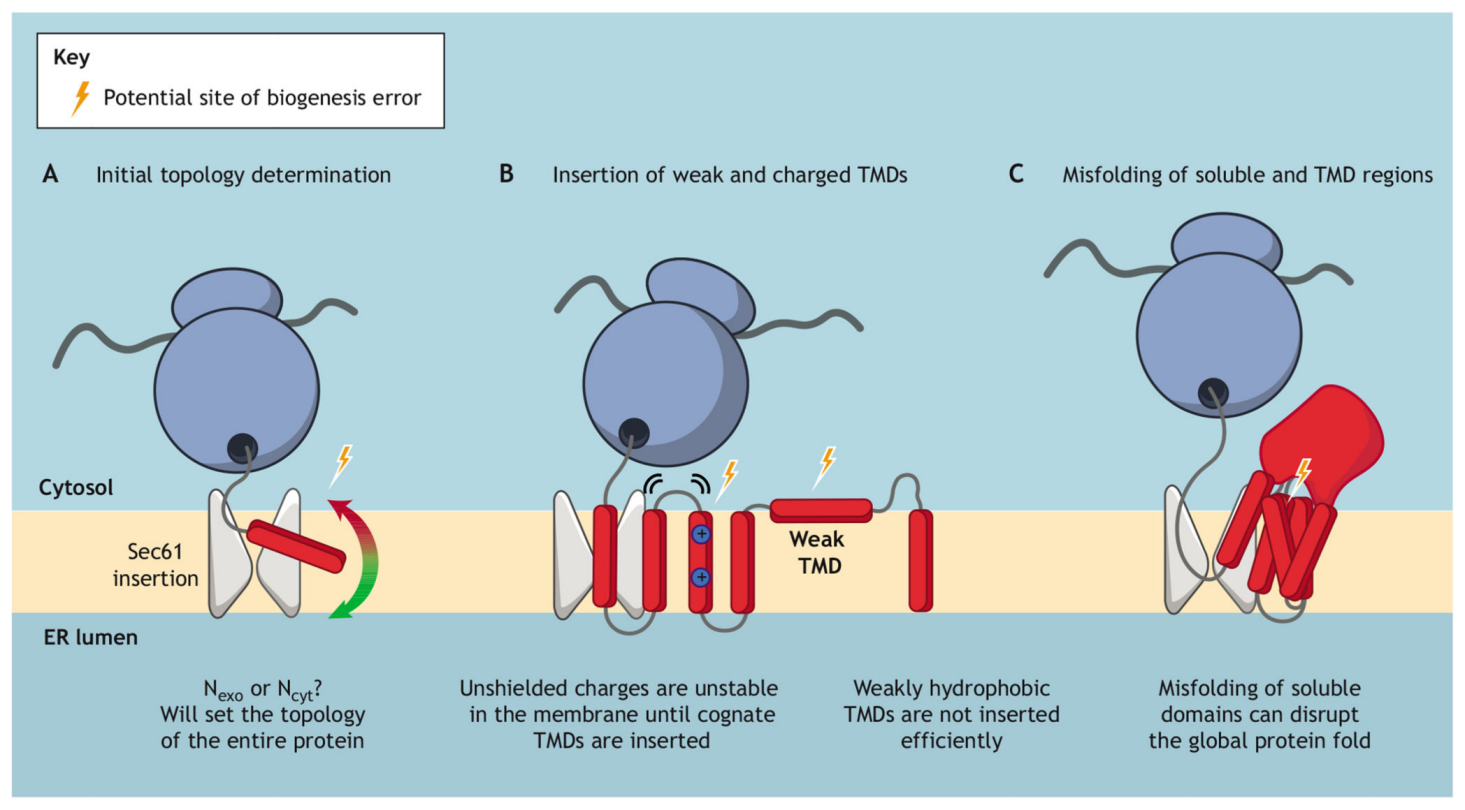
Potential sites of error during co-translational insertion of membrane proteins at the ER. Membrane protein biogenesis at the ER begins with targeting of a translating nascent chain to an hour-glass shaped channel called the Sec61 translocon. The nascent chain is inserted into the Sec61 translocon and then partitions into the phospholipid bilayer through a lateral gate. During this process there are several instances where errors may arise. (A) Orientation of the first TMD as it enters the membrane, where the N-terminus must be correctly localised to the ER lumen (N_exo_) or cytoplasm (N_cyt_). This orientation defines the topology of the subsequent TMDs, meaning errors at this stage can result in incorrect topology of the entire protein. (B) Failed insertion of poor TMDs. Some TMDs are weakly hydrophobic or charged and, as a result, may not insert correctly in the absence of their cognate binding TMDs. (C) Misfolding of soluble domains. In some instances, MPs contain large soluble domains, which are required to fold correctly for overall protein fold. Failure to fold such domains can therefore also impact TMD packing.

In this co-translational insertion process, there are multiple opportunities for errors to arise and, as a result, several potential points where quality control might be required (Fig. 1). The first potential source of error lies in establishing the correct topology during insertion of TMDs (Coelho et al., 2019). The topology decision for the first TMD is particularly important as it usually determines the topology of the entire protein, with some notable exceptions (Gafvelin et al., 1997; Sato et al., 1998a,b; Lu et al., 2000). Establishing the correct topology for a multi-pass MP has been likened to establishing the ‘reading frame’ during translation and is of paramount importance for correct biogenesis. A notable feature of MPs, with the exception of type 1 MPs, is that positive residues tend to be enriched in cytosolic sequences flanking the TMDs, leading to the ‘positive inside rule’ (Von Heijne, 1986). In bacteria, it is likely that this topology is partly dictated by the proton motive force across the membrane (Cao et al., 1995). In eukaryotic cells, the source of this discrimination is less clear, although charges on the cytosolic face of Sec61 and auxiliary translocon associated factors have been implicated (Goder et al., 2004; Higy et al., 2004).

A second potential source of error is the integration of TMDs into the bilayer. Many TMDs contain charged and polar residues that are essential for function, for example, in pore formation for ion channels (Tao et al., 2009) or membrane occlusion sites for transporter proteins (Kato et al., 2015; Hiraizumi et al., 2019). As a result, many TMDs are only weakly hydrophobic and their insertion into the membrane is not as favourable as the insertion of more hydrophobic TMDs (Enquist et al., 2009). Insertion defects are often magnified by disease-causing mutations (Schlebach and Sanders, 2015), and mutation of hydrophobic to polar residues in TMDs of MPs is disproportionately likely to cause disease (Partridge et al., 2004). In the final fold of a protein, polar and charged residues are normally protected from the hydrophobic core of the lipid bilayer through interactions with other TMDs. However, due to the sequential nature of TMD insertion, cognate interacting residues may not be available until all the TMDs are inserted, rendering unshielded residues vulnerable to reduced membrane partitioning. Indeed, intimate contacts between early and late emerging TMDs have been observed in structures such the cystic fibrosis transmembrane conductance regulator (CFTR) (Zhang and Chen, 2016). These contacts cannot be fulfilled until insertion of the final TMDs, so upstream TMDs may either fail to insert properly or require assistance for stable insertion until synthesis is complete. Furthermore, the broader protein fold may be destabilised in the absence of these favourable interactions. In support of the importance of these interactions, there is experimental evidence for force generation as a result of interactions between C-terminal and N-terminal TMDs of an MP during co-translational insertion (Cymer and Von Heijne, 2013).

Finally, MPs can contain large soluble domains, on both the lumenal and cytosolic sides of the membrane, that must also fold correctly. Mutations in such soluble domains can result in extensive misfolding throughout the protein, including in the TMDs. Such widespread misfolding is found in the most common diseasecausing mutation in CFTR, ΔF508 (De Boeck et al., 2014). This residue maps to the first cytosolic nucleotide-binding domain, but forms the basis of an important interface with the cytosolic loops that join the membrane-spanning helices, resulting in destabilisation of the entire protein fold when deleted (Mendoza et al., 2012). Mutations in either TMDs or soluble domains can impact protein folding during insertion into the ER membrane and therefore must be managed by a cellular quality control system.

## Co-translational quality control

Insertion, topology and TMD-folding challenges occur during translation of MPs. In contrast, the best-studied quality control systems (e.g. ERAD) act post-translationally. This temporal separation allows the nascent protein to engage with machinery that can promote folding prior to decisions about degradation. However, recent data suggest that an additional form of quality control can occur co-translationally (Lakshminarayan et al., 2020; Trentini et al., 2020). This co-translational process appears to be a form of quality control called ribosome-associated quality control (RQC). The RQC pathway, which degrades nascent polypeptide chains, along with the related non-stop decay (NSD) (Frischmeyer et al., 2002; van Hoof et al., 2002) and no-go decay (NGD) (Doma and Parker, 2006) mRNA-surveillance pathways, are triggered by problems that arise from aberrant mRNAs. Such problems can include mRNA lesions that prevent translation elongation and mRNAs that lack a stop codon before a poly-adenine [poly(A)] sequence (Joazeiro, 2019). Although RQC has predominantly been studied in the context of cytosolic proteins, emerging evidence suggests that similar pathways can also function at the ER (Brandman and Hegde, 2016; Joazeiro, 2019), and employ some of the core machinery that has been well characterised for the RQC-based recognition and resolution of cytosolic mRNA lesions. The term ER-RQC was coined to describe the action of this quality control process at the ER (Joazeiro, 2019).

RQC is triggered when translation elongation becomes stalled. The best studied example of a stall-inducing event is a ‘non-stop’ transcript, in which a poly(A) sequence occurs without an upstream stop codon. When a ribosome translates through a ‘non-stop’ poly(A) tract, charge interactions between the nascent chain and the ribosome exit tunnel result in a slowdown of translation across approximately six codons, caused by changes to the geometry of the peptidyl-transferase centre (PTC) (Chandrasekaran et al., 2019; Tesina et al., 2020). This translational slowing eventually leads to stalling, which is ultimately caused by single-helix stacking interactions between the mRNA and ribosomal RNA that perturb elongation by sterically excluding the formation of the tRNA–eEF1A–GTP ternary complex that is required for elongation.

The cue that activates RQC of the nascent protein is a collision between an upstream ribosome and the terminally stalled downstream ribosome (Juszkiewicz et al., 2018; Ikeuchi et al., 2019a,b). The collided state forms a unique interface, which is recognised by the E3 ubiquitin ligase ZNF598 (Hel2 in yeast). ZNF598 ubiquitylates uS10, uS3 and eS10 on the small ribosomal subunit; this signals commitment to the RQC pathway and initiates recruitment of additional factors that drive ribosome recycling and degradation of the nascent chain (Garzia et al., 2017; Juszkiewicz and Hegde, 2017; Matsuo et al., 2017; Sitron et al., 2017; Sundaramoorthy et al., 2017). The machinery involved in these downstream processes are well described, although some details of engagement remain incompletely understood. Stalled ribosomes with a codon in the A site are split by the activating signal cointegrator 1 complex (ASCC) complex (comprising ASCC1, ASCC2 and ASCC3) (Juszkiewicz et al., 2020a,b) in mammals or the homologous RQT complex (Matsuo et al., 2017; Sitron et al., 2017) in yeast [Slh1 and Cue3 (also known as Rqt2 and Rqt3, respectively), and Rqt4] (Matsuo et al., 2020). In addition, a complex comprising Dom34 and Hbs1 (Pelota and Hbs1 plus ABCE1 in mammals) splits ribosomes stalled at truncated mRNAs with an empty A site (Shoemaker et al., 2010; Pisareva et al., 2011; Tsuboi et al., 2012) in a process that can act independently of ZNF598. Once the ribosome has been split, the exposed tRNA bound to the 60S ribosomal subunit is recognised by Rqc2 and the E3 ubiquitin ligase Ltn1 (NEMF and listerin in mammals), which ubiquitylate the nascent chain, marking it for degradation at the proteasome (Chu et al., 2009; Bengtson and Joazeiro, 2010; Brandman et al., 2012; Shao et al., 2013). The nascent chain is then liberated from the tRNA via the action of Vms1 (ANKZF1 in mammals) (Izawa et al., 2017; Verma et al., 2018; Su et al., 2019; Yip et al., 2019) and extracted from the exit tunnel before degradation at the proteasome in a process dependent on Cdc48 (Brandman et al., 2012; Defenouillère etal., 2013). In *Saccharomyces cerevisiae*, Hel2-mediated ubiquitylation is also a trigger for degradation of the mRNA via the 5′-3′ exonuclease Xrn1 (Tesina et al., 2019), the endonuclease Cue2 (D’Orazio et al., 2019) and the 3′-5′ exonuclease activity of the cytoplasmic exosome, which is recruited to the ribosome by the Superkiller (SKI) complex (van Hoof et al., 2002). It is likely that mammals degrade the mRNA in a similar manner, although this is less well characterised experimentally. An additional strand of NGD has recently been described in *S. cerevisiae* that is distinct from the pathway that engages traditional RQC. In this pathway, termed NGD^RQC^, Hel2-mediated ubiquitylation is preceded by Not4-mediated mono-ubiquitination of the ribosomal protein eS7 (Ikeuchi et al., 2019a,b) resulting in endonucleolytic mRNA cleavage upstream of the collided disome.

Rqc2 (NEMF in mammals) performs a second function in RQC in *S. cerevisiae* by catalysing the extension of the nascent polypeptide chain into C-terminal alanine-threonine (CAT) tails (Shen et al., 2015). This protein synthesis reaction is functionally independent of the 40S ribosomal subunit or any of the traditional translational GTPases (Osuna et al., 2017), and acts to push the nascent chain out of the ribosome exit tunnel. This extension is thought to serve two main purposes. First, it increases the likelihood of an available ubiquitin acceptor lysine residue coming into contact with the Ltn1 catalytic domain, which resides at the mouth of the ribosomal exit tunnel (Shao et al., 2015; Kostova et al., 2017) to ubiquitylate substrates. Second, the resulting CAT tail induces the aggregation and potentially also degradation of substrates (Yonashiro et al., 2016; Izawa et al., 2017; Sitron and Brandman, 2019). A form of CAT-tailing has recently been discovered in bacteria, indicating that it may be one of the most ancient forms of co-translational quality control (Lytvynenko et al., 2019). Recent reviews have covered the process of soluble RQC in extensive detail (Ikeuchi et al., 2019a,b; Joazeiro, 2019), instead we shall focus on the newly discovered RQC of MPs.

## ER-RQC of MPs

**Fig. 2:**
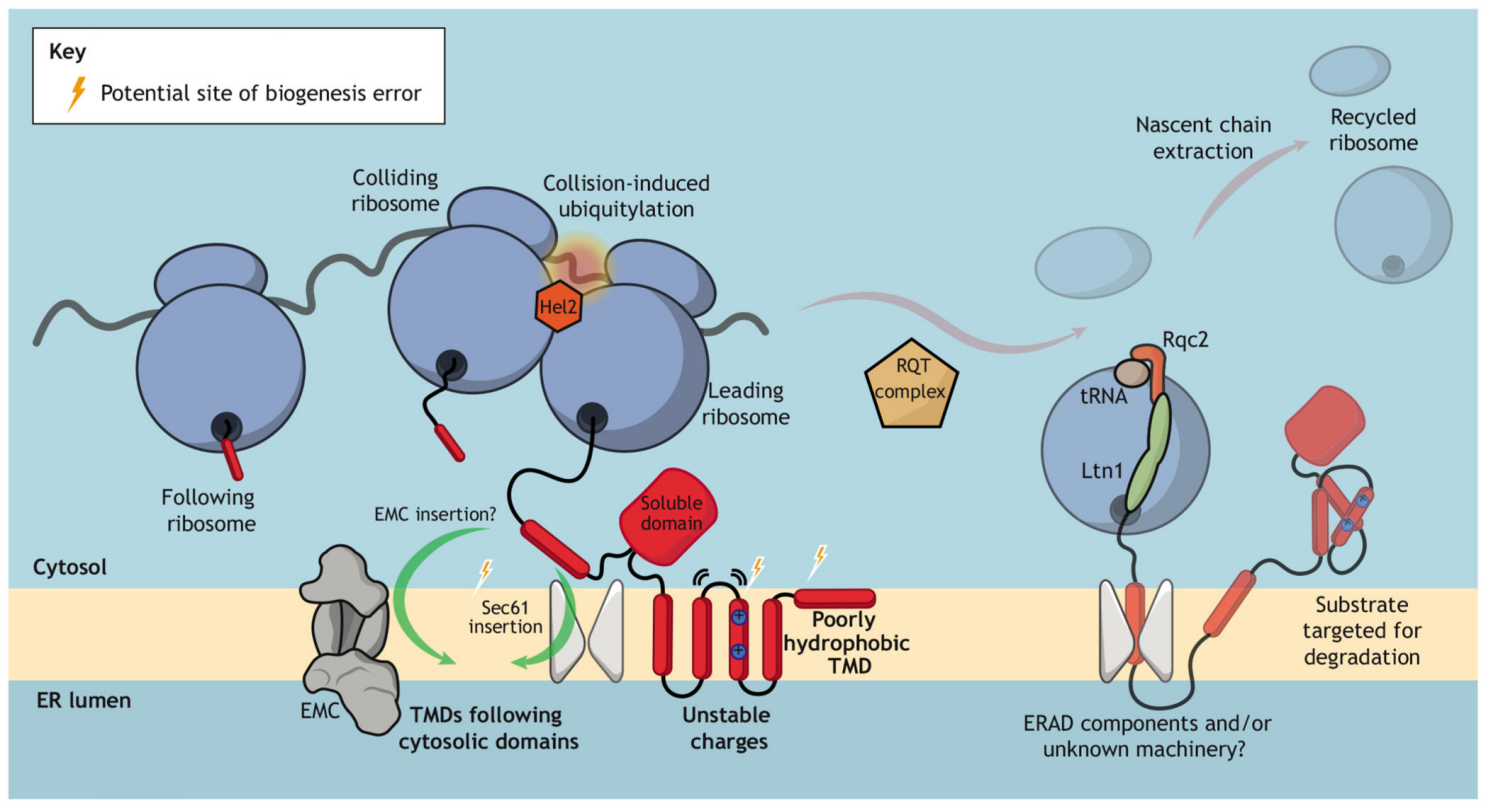
Membrane protein biogenesis defects result in ER-RQC. Defects in TMD insertion through the EMC or Sec61, attempted insertion of poorly hydrophobic TMDs or poor shielding of TMD charges might all contribute to ribosome stalls. Additionally, insertion of TMDs after large soluble domains might also present a challenge due to release of the ribosome from the translocon while cytoplasmic domains are synthesised. Translational stalls at the ER that result in ribosome collisions are recognised by the ubiquitin ligase Hel2, followed by splitting of the ribosome by the RQT complex (and potentially also Dom34-Hbs1). Following splitting of the ribosome, the exposed nascent chain-tRNA complex is recognised by Rqc2 and listerin (Ltn1). This complex ubiquitylates the nascent chain, targeting it for degradation. The final step of extraction and degradation of the chain is less well understood, and evidence concerning details of this process at the ER is sparse. Extraction and degradation of substrates at the ER may involve as yet unidentified proteins, potentially including ERAD machinery.

RQC events at the ER have largely been studied in the context of aberrant mRNAs that are similar to well-defined cytoplasmic substrates but directed to the ER (see Box 1). Insights obtained from two new studies now specifically link the biogenesis of large multipass MPs with RQC (Lakshminarayan et al., 2020; Trentini et al., 2020) in the absence of obvious mRNA defects. Investigating the biogenesis of the multi-pass yeast ABC transporter Yor1, our laboratory, together with collaborators, discovered that misfolded Yor1 was subject to co-translational degradation, dependent on the specific type of misfolding induced by different mutations (Lakshminarayan et al., 2020). This quality control event was independent of ERAD genes but could be rescued by deletion of Hel2 and other RQC factors. Furthermore, degradation was also attenuated by reducing ribosome density on mRNAs or by increasing the surface area of the ER membrane (see Box 2) (Schuck et al., 2009; Lakshminarayan et al., 2020). Taken together, these findings support a role for ribosome collisions in the degradation process (Lakshminarayan et al., 2020). Such a discovery marks a departure from previous studies of ER-RQC (see Box 1), as it suggests that the biogenesis state of the protein can also act as an input to RQC. As such, the RQC machinery is sensitive not just to the hard-coded information in the mRNA, but also to dynamic cues from the folding nascent chain. In a second study, listerin-knockout human cells were used to demonstrate that RQC contributes to generation of antigenic peptides (Trentini et al., 2020). Importantly, immunopeptidome analysis revealed that multipass MPs are specifically enriched as a class of listerin-dependent substrates. One implication of this enrichment is that MPs constitute a significant portion of so-called defective ribosomal products, or DRiPs, that are a source of self-peptides, and likely reflect errors in MP biogenesis (Trentini et al., 2020). Collectively, these observations suggest that aberrant biogenesis of MPs can trigger ER-RQC (Fig. 2). Intriguingly, co-translational ubiquitylation of CFTR and apolipoprotein B100 were first observed over 20 years ago (Sato et al., 1998b; Zhou et al., 1998), but it has only recently been realised that this may be the result of RQC.

Notably, both recent studies of ER-RQC of MPs implicate the ER membrane complex (EMC), a conserved complex of 6—10 ER membrane and cytoplasmic proteins (Jonikas et al., 2009; Wideman, 2015). The EMC can act as an insertase for tail-anchored (TA) proteins (Guna et al., 2017) and transmembrane proteins that have short lumenal N-termini (N_exo_ topology) (Chitwood et al., 2018). Recent structural characterisation of the EMC has suggested that it could mediate the insertion of hydrophobic TMDs through a mechanism that reduces the energetic barrier of TMD insertion into the membrane (Bai et al., 2020; O’Donnell et al., 2020; Pleiner et al., 2020). However, the EMC has been widely implicated in the biogenesis of many MPs that do not assume an Nexo or TA topology (Luo et al., 2002; Bircham et al., 2011; Christianson et al., 2012; Louie et al., 2012; Richard et al., 2013; Satoh et al., 2015; Bagchi et al., 2016; Marceau et al., 2016; Tang et al., 2017; Volkmar et al., 2018; Barrows et al., 2019; Tian et al., 2019; Xiong et al., 2020; Chitwood and Hegde, 2019; Hiramatsu et al., 2019). The broad range of topologies of EMC substrates has led to suggestions of a role as a co-translational intra-membrane chaperone (Shurtleff et al., 2018; Coelho et al., 2019; Hiramatsu et al., 2019; Lin et al., 2019; Ngo et al., 2019; Volkmar and Christianson, 2020), although a chaperoning function is yet to be functionally demonstrated. In the case of yeast Yor1-ΛF, which has its N-terminus in the cytoplasm (i.e. N_cyt_ topology), biogenesis defects were triggered by the combination of protein misfolding and deletion of EMC. Mutations that restrict the gating properties of the Sec61 translocon similarly triggered ER-RQC (Lakshminarayan et al., 2020). In human cells, where deletion of listerin revealed ER-RQC of multi-pass MPs, the EMC was upregulated, suggesting that induction of membrane insertion machinery is an adaptive response to the presence of aberrant MPs (Trentini et al., 2020). Together, these links to EMC and Sec61 suggest that ER-RQC can be triggered by errors in the insertion of TMDs at the ER, and that the machinery is responsive to folding states of nascent polypeptides rather than just mRNA lesions. This distinction is of particular note as, so far, RQC studies have been linked explicitly to errors in mRNA rather than in the biogenesis state of the polypeptide. The relative contributions of mRNA errors and protein biogenesis errors in RQC at the ER remains unclear and is an important area for future investigation (see Box 1). Intriguingly, mutation of hydrophobic residues in the TMD of the low density lipoprotein receptor (LDLR) with arginine residues results in degradation that is independent of ERAD or the lysosome, potentially also as a result of ER-RQC (Strøm et al., 2017).

Plants and mammals also possess an ER-associated quality control pathway that involves UFMylation of collided ribosomes followed by autophagic trafficking of substrates to the lysosome (Madlen et al., 2020; Walczak et al., 2019; Wang et al., 2020). Although this pathway has been characterised with artificially stalled substrates, proteomic analysis suggests many potential clients accumulate when this pathway is perturbed, suggesting a broad spectrum of potential targets.

## Characteristics of ribosome stalls during MP biogenesis

The nature of ribosome-stalling events that trigger ER-RQC of MPs are likely to be distinct from the well-characterised examples of cytoplasmic RQC. ER-RQC of yeast Yor1 seems sensitive to MP length, folding state and the function of the insertion machinery (Lakshminarayan et al., 2020). Cytoplasmic RQC systems are likely tuned to prevent the dissolution of ribosomes that have merely slowed in translation but are not permanently arrested (Hickey et al., 2020; Juszkiewicz et al., 2020a). Such fine tuning is important, as translation rates are known to vary significantly across the genome (Fluitt et al., 2007; Darnell et al., 2018; Wu et al., 2019), and such variation is essential for many aspects of protein biogenesis (Nedialkova and Leidel, 2015; Yu et al., 2015). Tolerance to translational slowing is probably even more important in the context of secretory and MPs, where translation likely slows in response to SRP recruitment (Pechmann et al., 2014; Schibich et al., 2016), increasing the available time for targeting to the ER. As moderate translational slowing can favour biogenesis and presumably does not induce ER-RQC, it seems likely that the stalls that trigger the quality control checkpoint are more complex than a moderate slowing of elongation that leads to ribosome collision.

It remains unclear how MP biogenesis influences translation kinetics; however, examples from the diverse range of structurally characterised stalling sequences may provide some clues. Examples of such stalling sequences, or arrest peptides, include MifM and SecM in bacteria (Chiba and Ito, 2012; Gumbart et al., 2012), the gp48 uORF2 sequence employed by human cytomegalovirus (hCMV) (Bhushan et al., 2010), and stalling of the unspliced form of the UPR activator XBP1 (XBP1-u) at the ER membrane in eukaryotes (Schibich et al., 2016). In each case, stalling is facilitated by interactions between the nascent chain and the ribosome exit tunnel. Although the specific mechanisms of these different stalling events are highly heterogenous, the stalled intermediates appear to share some features in common (Wilson et al., 2016): (1) intimate interactions between the backbone and the ribosome exit tunnel; (2) abnormal or unexpected peptide bond conformations in the nascent chain near the peptidyl-transferase centre (PTC); and (3) altered conformations of key amino acids and nucleotides surrounding the PTC. In general, interactions between the nascent chain and the ribosome exit tunnel, and abnormal geometries around the PTC are key features in a variety of stalling sequences.

Ribosome exit tunnels must accommodate translation of the entire cellular proteome (Dao Duc et al., 2019) while avoiding significant interaction with translocating nascent chains. However, some sequences are more prone to stable interactions than others. Of particular relevance to MP biogenesis, the N-terminal signal peptide of the secreted protein PCSK9 is prone to stalling the ribosome through interactions with the walls of the ribosome exit tunnel (Li et al., 2019; Liaud et al., 2019). It is possible, therefore, to envisage TMDs, which are hydrophobic and prone to helix formation within the ribosomal exit tunnel (Bañó-Polo et al., 2018), to be particularly inclined to slow movement leaving the ribosome. Such a hypothesis is analogous to that of uORF2 of hCMV gp48, where the stalling sequence forms a helix in the ribosome exit tunnel proximal to the PTC, which consequently disrupts the geometry of the PTC, inhibiting conventional translation termination (Matheisl et al., 2015). Similarly, a structure of the first example of an endogenous RQC substrate, *S. cerevisiae* Sdd1, revealed important hydrophobic interactions with the ribosomal exit tunnel, helix formation and an altered conformation of the PTC (Matsuo et al., 2020).

**Fig. 3:**
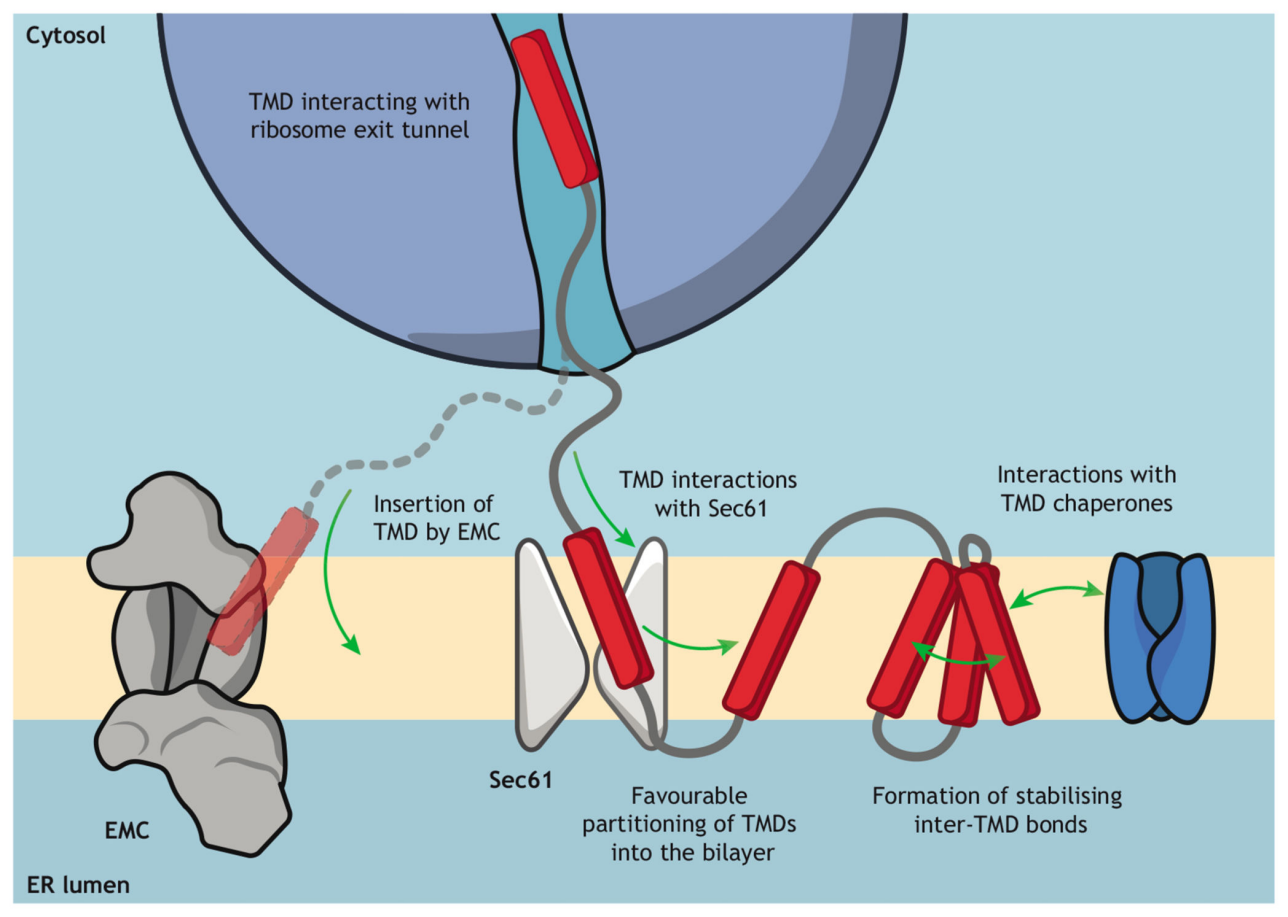
Sources of tension to overcome ribosome stalls. Interactions between nascent TMDs and the ribosomal exit tunnel may result in a slowing down in translation. There are multiple sources of tension that may provide the force on the nascent chain required to overcome this type of stall. Tension may be generated by any of the following: interactions between the nascent chain and the Sec61 translocon, favourable partitioning of a TMD into the lipid bilayer, action of the EMC (either as an insertase or as a chaperone), inter-TMD interactions within the MP, or interactions between TMDs and a chaperone.

Another factor pertinent to induction of ER-RQC is the force generated by normal insertion of TMDs into the lipid bilayer (Cymer et al., 2014; Niesen et al., 2018). Tension on the nascent chain could be generated by favourable interactions between substrates and the Sec61 translocon, EMC or other insertases, and chaperones. In addition, the energetically favourable partitioning of hydrophobic TMDs into the bilayer may create a pulling force on the nascent chain (Fig. 3). Although such proposed models are speculative in the absence of empirical data on the role of nascent chain tension in ER-RQC (discussed further below), such tension could help overcome potential resistance generated by interactions between TMDs and the ribosome exit tunnel. As such, the favourable insertion of TMDs might offset any ‘sticky’ interactions in the exit tunnel and help to avoid ribosome collisions. This model is consistent with the observation that mutations affecting the co-translational insertion of TMDs by the Sec61 translocon induce ER-RQC of yeast Yor1 ΔF (Lakshminarayan et al., 2020). Alternatively, it is possible that mutations in Sec61 create a physical ‘roadblock’ (Lakshminarayan et al., 2020), which would sterically prevent access of substrates to the membrane, leading to translational stalling. It is clear that there are several potentially viable explanations for the mechanism of misfolding-induced ER-RQC.

Furthermore, precisely how the EMC might contribute to avoiding translational pausing and ribosome collision remains to be seen. The EMC is required for the biogenesis of Nexo and many TA MPs (Guna et al., 2017; Chitwood et al., 2018; Volkmar et al., 2018; Chitwood and Hegde, 2019), but it is not clear whether EMC is directly involved in biogenesis of MPs that do not assume the N_exo_ topology, or whether observed synthesis effects are the result of loss of another, as yet unidentified, EMC client. It is possible that the EMC is required for the correct insertion of weak initial TMDs, which are missed by SRP, even if they are not in the N_exo_ orientation. Similarly, the EMC may be required to re-insert TMDs that are weakly hydrophobic and fail to insert in the absence of their cognate binding TMD (Chen and Zhang, 1999) (Fig. 2). Accordingly, loss of EMC function in the context of diseasecausing hydrophilic mutations in the TMDs of connexin-32 exacerbates failed integration of internal TMDs into the bilayer (Coelho et al., 2019).

Finally, a related means of stalling is achieved by bacterial arrest peptides that trigger stalling on membrane-bound ribosomes in a mechanism that may be relevant for ER-RQC MP substrates (Wilson et al., 2016). Here, the ribosome is targeted to the membrane but translation stalls in the absence of an active pulling force provided by the SEC translocation machinery. The force provided by the SEC machinery allows the ribosome to overcome a stall caused by the compact α-helical structure of the arrest peptide in the exit tunnel, which otherwise disrupts the peptidyl-transferase activity of the ribosome (Sohmen et al., 2015; Zhang et al., 2015; Su et al, 2017). It is thus possible that monitoring tension on the nascent chain during membrane translocation or insertion is an ancient method for enforcing regulation of biogenesis and quality control at cellular membranes.

## Conclusions and perspectives in ER-RQC

The recent identification of folding-sensitive ER-RQC of multispanning MPs raises several key questions. First of all, it is unclear whether the canonical RQC machinery is sufficient for this form of ER-RQC or whether additional proteins are involved. In order to define the minimal machinery for ER-RQC, it will be necessary to identify tractable substrates for ER-RQC, akin to the fluorescent poly(A) reporters widely utilised in the study of cytosolic RQC (Juszkiewicz and Hegde, 2017). Model substrates will enable genetic screening to identify and characterise components that are specific to RQC at the ER. One challenge in such an approach is likely to be redundancy between RQC and collision-induced mRNA decay, since early experiments indicate that the two processes can be synergistic at the ER (Arakawa et al., 2016). Additional redundancy with ERAD components may also be a complicating factor. Although degradation of Yor1-ΔF was independent of known ERAD genes (Lakshminarayan et al., 2020), other studies have implicated ERAD machinery in the extraction of RQC substrates at the ER(Cesaratto et al., 2019). Such differences might indicate substrate specificity, further complicating the use of generic reporters. Thus, it will be important to, in parallel, determine the native substrate pool for ER-RQC, building on existing data that identify some substrates potentially prone to ER-RQC (Arakawa et al., 2016). Novel approaches utilising sucrose-gradient fractionation and quantitative mass spectrometry have recently been used to identify the co-translational regulator EDF1, which acts at collided ribosomes (Sinha et al., 2020). Similar approaches may also prove valuable in investigating RQC at the ER.

It remains to be established how certain specific pauses avoid recognition by the RQC machinery; for example, SRP-mediated translational slowing. Although the extent of stalling induced by SRP binding to the ribosome is unclear in living cells (Pechmann et al., 2014; Schibich et al., 2016), it likely results in at least some slowing down of translation. Whether or not additional machinery is required to specifically shield SRP-bound ribosomes from RQC is unclear. Similarly, the relative interplay between the UPR and XBP1-u regulation via RQC (Han et al., 2020) remains to be explored, as well as how ER-RQC integrates into the cellular proteostasis network more broadly. Hartl and colleagues note that one potential reason for increased ribosome stalling at the ER could be the non-specific nuclease activity of Ire1 α (also known as ERN1) (Trentini et al., 2020), which can produce truncated transcripts at the ER that would ultimately require rescue via RQC. Non-specific mRNA cleavage by Ire1 α has been demonstrated to form an integral part of the protective proteostasis network and has been termed regulated Ire1-dependent decay (RIDD) (Hollien and Weissman, 2006; Hollien et al., 2009). By degrading mRNAs destined for insertion at the ER, RIDD relieves pressure on folding and insertion resources at the ER during acute ER stress. RQC pathways would be necessary to resolve ribosomes that translate to the end of a truncated mRNA with no stop codon. Such a potential relationship between RIDD and ER-RQC substrates merits further investigation, especially considering the significance of Ire1 function in human disease (Maurel et al., 2014; Yan et al., 2019).

One final area of interest with respect to ER-RQC is the interplay between productive protein folding and the avoidance of ribosome stalls. Single-molecule experiments have recently revealed the folding trajectory of a multi-pass MP at the scale of helix-bundle formation (Jefferson et al., 2018; Choi et al., 2019; Krainer et al., 2019). In models of misfolding-induced RQC, it will be important to establish the potential sources of force generation at multiple stages of MP biogenesis. Folding measurements of ER-RQC substrates in the presence or absence of potential modifiers, such as the EMC, will help dissect the contribution of insertases and chaperones in TMD insertion and folding. Such an approach has been previously used to probe the role of the EMC3 homolog YidC in the folding of the LacY MP (Serdiuk et al., 2016). These experiments can be conducted under tightly regulated conditions using lipid nanodiscs, allowing precise control over relative stoichiometries and lipid composition. As such, they provide a unique opportunity to directly measure the contribution of probiogenesis factors in the absence of confounding cellular machinery. However, it will also be necessary to investigate force generation in relation to RQC and MP biogenesis in the cellular context. Arrest peptides can be finely tuned to function as force sensors (Cymer et al., 2015a), which will be useful in studying force generation during MP insertion. Force sensors could be used to probe the impact of disease-causing mutations, defined ER-RQC substrates and EMC substrates. Such approaches have been used previously to investigate force generation during insertion by the Sec61 translocon (Ismail et al., 2015) and during both conventional MP folding (Cymer and Von Heijne, 2013) and soluble protein folding (Farías-Rico et al., 2018).

Clearly there is much future work to be done to expand on our understanding of the scope, role and mechanism of ER-RQC in cells. In order to maximise efficiency of protein biogenesis while protecting against proteotoxicity, the cell must identify and neutralise the threats posed by misfolded proteins as early as possible. ER-RQC complements the variety of kinetic filters employed by membrane targeting machinery (Guna and Hegde, 2018) to ensure high fidelity in the earliest stages of integral MP biogenesis.

## Box 1 ER-RQC

RQC in the cytosol monitors the translation state of a ribosome to give a readout of the quality of the translated product. Because mRNA-dependent RQC should be agnostic to the location of the protein encoded by the mRNA, it likely occurs at comparable frequencies at the membrane and in the cytosol. The fact that almost a quarter of nascent proteins are produced at the ER (Fagerberg et al., 2010) suggests that MPs and soluble proteins that are translocated into the ER lumen account for a significant fraction of total cellular RQC substrates. However, RQC of membrane and secretory proteins presents a distinct set of challenges, since stalled peptides may need to be extracted both from the ribosome and the membrane and/or translocon prior to degradation. Artificially induced stalls on a truncated nascent chain demonstrated that listerin and NEMF can function at the ER membrane (Von Der Malsburg et al., 2015) and are not sterically impeded in accessing substrates as they ‘backslide’ out of the translocon. Additional studies support a role for Dom34–Hbs1 in the degradation of non-stop constructs targeted to the ER or mitochondria, with the substrate either released into the lumen or degraded (Izawa et al., 2012; Crowder et al., 2015; Arakawa et al., 2016). Although these studies demonstrated that RQC can occur at the ER, it was still considered to mostly be triggered by inappropriate polyadenylation (Ozsolak et al., 2010; Pelechano et al., 2013; Chandrasekaran et al., 2019), which is presumed to be broadly agnostic to the specific biology or cellular localisation of substrates. More recently, the regulatory XBP1-u mRNA(Ron and Walter, 2007) has been revealed as a target of RQC at the ER (Han et al., 2020). The relative contribution of aberrant mRNAs compared to misfolding-driven RQC at the ER is an important area for further study.

## Box 2 Constraints influencing ribosome collisions on the membrane

An important question in ER-RQC is how the recruitment of ribosomes to the ER membrane influences the likelihood of collisions and induction of quality control events. Early modelling suggested that the arrangement of the co-translational insertion machinery still permitted access of listerin to the nascent chain (Von Der Malsburg et al., 2015), and it was later suggested that the collided disome structure could still form stably at the membrane if both ribosomes were bound to a minimal Sec61 translocon complex (Juszkiewicz et al., 2018). The potential steric impact of translocon-associated accessory factors, such as the oligosaccharyl transferase (OST) complex (Braunger et al., 2018) and others (McGilvray et al., 2020), remains to be explored. It is clear that a stable collided disome structure requires significant bending of the ER membrane, though not beyond levels of curvature regularly observed in electron micrographs. It is even less clear how tethering translating ribosomes to the membrane surface impacts the likelihood of collisions occurring. It is possible that binding translating ribosomes to Sec61 channels reduces the chance of collisions occurring compared to cytosolic ribosomes, by introducing an extra level of spatial restriction. The only clue regarding the impact of translocon spacing on ER-RQC comes from experiments where a large expansion of the ER was triggered by the deletion of *OPI1*, a negative regulator of ER lipid biosynthesis. This expansion does not increase the abundance of the Sec61 translocon (Schuck et al., 2009). As a result, the translocons are spaced further apart from each other, a condition that rescued the degradation of Yor1-ΔF, an ER-RQC substrate (Lakshminarayan et al., 2020). Because expansion of the ER can improve folding capacity independent of chaperone levels, it will be important to investigate the impact of translocon surface density on ER-RQC more rigorously in future.
